# Bone marrow mesenchymal stromal cell-derived small extracellular vesicles: A novel therapeutic agent in ischemic heart diseases

**DOI:** 10.3389/fphar.2022.1098634

**Published:** 2023-01-05

**Authors:** Wenguang Chang, Peifeng Li

**Affiliations:** Institute for Translational Medicine, The Affiliated Hospital, College of Medicine, Qingdao University, Qingdao, China

**Keywords:** BMMSC, heart failure, myocardial infarction, extracellular vehicles (EVs), ischemia, reperfusion

## Abstract

Myocardial injury is a major pathological factor that causes death in patients with heart diseases. In recent years, mesenchymal stromal cells (MSCs) have been generally used in treating many diseases in animal models and clinical trials. mesenchymal stromal cells have the ability to differentiate into osteocytes, adipocytes and chondrocytes. Thus, these cells are considered suitable for cardiac injury repair. However, mechanistic studies have shown that the secretomes of mesenchymal stromal cells, mainly small extracellular vesicles (sEVs), have better therapeutic effects than mesenchymal stromal cells themselves. In addition, small extracellular vesicles have easier quality control characteristics and better safety profiles. Therefore, mesenchymal stromal cell-small extracellular vesicles are emerging as novel therapeutic agents for damaged myocardial treatment. To date, many clinical trials and preclinical experimental results have demonstrated the beneficial effects of bone marrow-derived mesenchymal stromal cells (BMMSCs) and bone marrow-derived mesenchymal stromal cells-small extracellular vesicles on ischemic heart disease. However, the validation of therapeutic efficacy and the use of tissue engineering methods require an exacting scientific rigor and robustness. This review summarizes the current knowledge of bone marrow-derived mesenchymal stromal cells- or bone marrow-derived mesenchymal stromal cells-small extracellular vesicle-based therapy for cardiac injury and discusses critical scientific issues in the development of these therapeutic strategies.

## Introduction

Ischemic heart diseases (IHDs) are cardiac dysfunctions caused by acute myocardial infarction (AMI) or ischemia reperfusion injury. Ischemic heart injury is the leading cause of death in patients. Although advanced therapeutic strategies have been developed, such as percutaneous coronary intervention (PCI), stenting, and routine use of antithrombotic medical treatment (Keeley and Weaver, 1999; Reed et al., 2017; Johnston et al., 2018; Jia et al., 2020), patients with IHD are still admitted with congestive heart failure and cardiogenic shock after revascularization (Thiele et al., 2019; Zeymer et al., 2020). Sudden death in patients with IHD remains at a high level.

As a novel treatment method, stem cell therapy has attracted much attention for its regenerative effects (Vining and Mooney, 2017; Nourian Dehkordi et al., 2019; Zhou et al., 2022). Stem cells used in cardiac injury therapy include cardiac stem cells, induced pluripotent stem cells, cardiovascular progenitor cells, peripheral blood stem cells, mesenchymal stromal cells, and so on (Shafei et al., 2017; Rikhtegar et al., 2019). MSCs are considered to be suitable for the treatment of various diseases due to their high self-renewal and multilineage differentiation potential (Mushahary et al., 2018). Additionally, *in vivo* and *in vitro* models, MSCs express specific cardiomyocyte markers (such as connexin 43 and N-cadherin) (Fukuda and Fujita, 2005). Thus, MSCs are thought to be suitable to treat cardiac disease (Carbone et al., 2021). Interestingly, preclinical and clinical data indicated that the mechanism of MSC therapy relies on its paracrine function rather than its differentiation and renewal ability in diseased tissues (Caplan, 2017; Yang et al., 2021). MSC-derived secretome derivatives (conditioned medium or exosomes) showed better potential due to their easy quality control, safety and efficacy (Mendt et al., 2019). Therefore, research mainly focuses on the secretomic roles of MSCs. Small extracellular vesicles (sEVs) are the most studied secretomes of MSCs in recent years. In our review, we summarized clinical studies that used BMMSCs to treat acute myocardial infarction (AMI) or ischemia-induced cardiac failure and BMMSC-derived sEVs in ischemic heart disease therapy and gathered experimental and clinical evidence from recent years of using BMMSCs and secretome-sEVs. By comparing the similarities and differences between various studies, we hope to provide a future research direction for BMMSC therapy.

## Characterization and biomarkers of BMMSCs

MSCs can be derived from various tissues, such as adipose, brain, pancreas, liver, amniotic fluid, synovia, peripheral blood, muscle tissues and bone marrow (Wang et al., 2018; Camernik et al., 2019). However, there are distinct properties in different sources of MSCs. For example, comparative studies have shown that BMMSCs have lower IDO activity (an enzyme that inhibits T-cell activation) than adipose-derived MSCs (AT-MSCs) (Strioga et al., 2012; Hao et al., 2017). The mRNA expression of SDF-1 (a chemokine, also known as CXCL12) and VCAM-1 (an adhesion protein) was higher in BMMSCs than in AT-MSCs and umbilical cord-derived MSCs (UCMSCs) (Cortes-Araya et al., 2018). In addition, MSCs derived from bone marrow were shown to have a 5.9-fold higher migratory capacity than UCMSCs, which is a key factor in post-traumatic tissue repair (Shi et al., 2021). BMMSCs have become one of the most widely used sources in preclinical and clinical studies as they are easily obtained. In the laboratory, BMMSCs are mostly obtained *via* a colony-forming unit-fibroblast approach, in which raw unpurified bone marrow is directly seeded into plates or flasks. To verify the phenotype of MSCs, researchers have determined the positive expression of biomarkers such as CD73, CD90, and CD105 and the negative biomarkers CD34, CD11b, CD14, CD19, CD45, and CD79a in experiments (Chang et al., 2022a). However, there is still a lack of specific biomarkers to distinguish stem/progenitor cells from other remaining cells. In human adults, cells expressing Lin^−^ CD45^−^ CD271^+^, along with low expression or negative expression of CD140a, were shown to have a higher population of MSC stem/progenitor cells; however, in human fetal bone marrow and murine MSCs, CD140a was found to be positively expressed (Li et al., 2016). For murine BMMSCs, LepR^+^ was reported to have high expression (Zhou et al., 2014), in addition to high expression of CD140a and Sca-1 and negative expression of CD45 and TER119 (Morikawa et al., 2009). Additionally, genetically modified specific genes, such as Prx1-cre in mouse, could identified as biomarkers for BMMSCs (Ding and Morrison, 2013), how these different cell populations overlap and the potentially functional difference between those populations are still unclear.

Numerous studies have shown that BMMSCs are good therapeutic agents for various diseases, as they accelerate wound healing (Wu et al., 2007; Demir et al., 2021), modulate the immune response (Zhang et al., 2019; Xin et al., 2020), and exhibit antidiabetic (Hamza et al., 2017; Aali et al., 2020) and neuroprotective effects (Uccelli et al., 2011; Nakano et al., 2020). Many clinical trials are in the recruiting phase or phase Ⅰ/Ⅱ, in which BMMSC administration is used to treat various diseases, including myocardial infarction, amyotrophic lateral sclerosis (ALS) and Crohn’s disease. (Uder et al., 2018). Most of the trials showed promising improvements for the diseases, and no severe adverse effects were observed.

## Clinical trials using BMMSCs for ischemic heart disease

Most animal experiments and clinical trials using BMMSCs to treat ischemic heart injury have shown a global improvement in myocardial function. The improved heart function may occur through enhanced angiogenesis, inhibited apoptosis of cardiomyocytes, and ameliorated inflammation and scar formation after MSC transplantation (Yu et al., 2017). However, in clinical trials, the results are inconsistent. For example, a randomized, single-blind, controlled clinical trial conducted on patients with ST-segment elevation myocardial infarction showed that autologous BMMSC transplantation by intracoronary delivery at the time of PCI did not promote the recovery of left ventricular function and myocardial viability in the following 6th or 12th month of follow-up (combined with the optimum medical treatment) (Kim et al., 2018) (NCT04421274). However, another clinical trial with a similar procedure indicated improved LV function in the 4th or 6th month of follow-up (Zhang et al., 2021). In addition, previous systematic reviews and meta-analyses have shown divergent results (Chugh et al., 2009; de Jong et al., 2014; Liu et al., 2014; Fisher et al., 2015; Lalu et al., 2018). Although many clinical trial results using BMMSCs as therapeutic agents for acute ischemia showed good responses in improving cardiac function, mortality and heart attacks and/or heart failure requiring rehospitalization following treatment (Xu et al., 2017; Attar et al., 2021), the systematic analysis indicated that this treatment may not lead to improvement when considering the “risk of bias” of trials, whether in the short term or the long term (Fisher et al., 2015) (Table 1). However, for chronic ischemic heart disease, a systematic analysis from the same group showed that BMMSC treatment may reduce the risk of long-term mortality in patients (Fisher et al., 2018), which is consistent with other reviews (Wen et al., 2011a; Xu et al., 2014). However, the effects of reduced mortality are not consistent in different studies (Fisher et al., 2016; Fu and Chen, 2020), (Table 1) suggesting unstable therapeutic effects of BMMSC treatment. In addition, the different MSC dosages affected the efficiency of BMMSCs. The optimal *in vivo* cell number for BLI and MRI was determined to be 1 × 10^6^ (Qu et al., 2022). An MSC dose of 10^7^–10^8^ cells was more likely to achieve better clinical endpoints, and the optimal time window for cell transplantation might be within 2–14 days after PCI (Yu et al., 2021). However, another analysis showed that patients exhibited an LVEF improvement with an MSC dose of less than 10^7^ cells combined with a transplantation time within 1 week (Wang et al., 2017). In addition to the dose controversy, methodologies for cell preparation also have impacts on the prognosis of AMI patients. A systematic review on methodology showed that nonuse of serum or plasma in the cell suspension is associated with a greater reduction in infarct size and a lower risk of all-cause mortality, and heparin usage could diminish the benefit in reducing IS (Yang et al., 2018). Therefore, a well-designed randomized control trial with unified cell preparation and administration doses, as well as rigorous evaluations of cardiac function and long-term clinical outcome follow-up, are required to further establish a clear risk-benefit profile of MSCs. However, there is growing evidence that BMMSC therapeutic effects might be indirect. Paracrine factors of BMMSCs, such as cytokines, miRNAs and exosomes secreted from stem cells, play a major role in the paracrine effects of stem cells (Wen et al., 2011b).

**TABLE 1:** Systematic clinical review of using BMMSC for ischemia heart disease treatment.

Review methods	Ischemic heart diseases focusing on	Clinical trial numbers included in analysis	Preliminary findings	Ref./year
Meta-analysis	Acute MI	41	No effect on morbidity, quality of life/performance or LVEF measured	Fisher et al. (2015)
Meta-analysis	Acute MI/IHD	23 (11 AMI and 12 IHF)	Improve LVEF in AMI patients, no difference in mortality	Lalu et al. (2018)
Meta-analysis	AMI	22	bone marrow-derived mononuclear cell/no effects	de Jong et al. (2014)
Meta-analysis	AMI	8	Improve LVEF by 3.17%	Liu et al. (2014)
Meta-analysis	AMI	13	Increases LVEF	Attar et al. (2021)
Meta-analysis	AMI	34	BMC transfer at 3–7 days post-AMI improve LVEF and decreasing LVESD or LVEDD.	Xu et al. (2017)
Meta-analysis	IHD	19	Improved LVEF and LVESV; No significant improvement in LVEDV	Xu et al. (2014)
Meta-analysis	IHD	8	Improve cardiac function and quality of life	Wen et al. (2011a)
Meta-analysis	IHD/congestive heart failure	38 (14 chronic IHD, 17 congestive HF and 7 intractable angina)	Reduced the incidence of long-term mortality, no effects on LVEF	Fisher et al. (2016)
Meta-analysis	IHD	6	Improve LVEF, no effects on mortality	Fu and Chen, (2020)
Meta-analysis	AMI	9	Increase in LVEF with a limited impact on LV volume and rehospitalization caused by HF.	Yu et al. (2021)
Meta-analysis	AMI	8	<10^7^ MSC within 1 week for AMI after PCI might improve LV function	Wang et al. (2017)
Meta-analysis	AMI	24	Methodological difference in cell transplantation have an impact on the cardiac parameters of patients	Yang et al. (2018)

AMI, acute myocardial infarction; IHD, Ischemic heart failure; PCI, percutaneous coronary intervention; MI, Myocardial infarction; LV, left ventricular; LVEF, left ventricular ejection fraction; LVEDD, left ventricular end-diastolic diameter; LVESD, left ventricular end-systolic diameter; LVESV, left ventricular end-systolic volume; LVEDV, left ventricular end-diastolic volume.

## sEV biogenesis and identification

**FIGURE 1:**
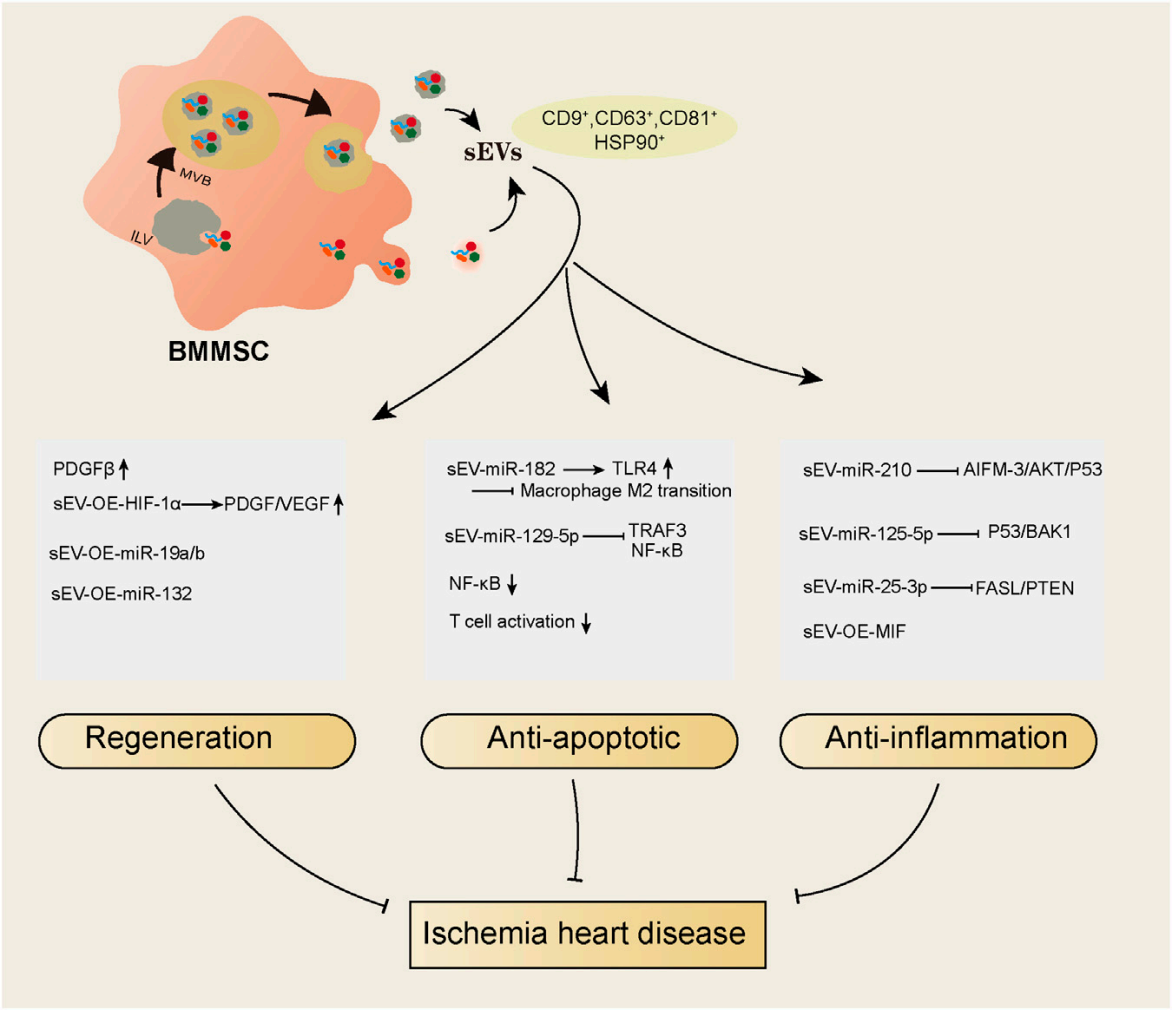
An overall summary of the IHD protective effects of sEVs derived from BMMSC.

EVs are double-membrane vesicles that are released into extracellular spaces by various types of cells. The classification of EVs depends on the size or biogenesis pathway or specific markers on the vesicles (van der Pol et al., 2012). By the biogenesis pathway, vesicles derived from the endosomal pathway are called exosomes, and vesicles derived from the plasma membrane budding pathway are called microparticles or microvesicles. By size, vesicles with diameters larger than 150 nm are called large extracellular vesicles, and vesicles with diameters between 50–150 nm are called small extracellular vesicles (sEVs) (Gould and Raposo, 2013; Wetzel, 2020). To date, most studies use the term “exosomes” to classify vesicles that have a size distribution of approximately 50–150 nm and positively express protein markers such as CD9, CD81, CD63, TSG101, flotillin and HSP90 (Chang et al., 2022b). However, these characteristics do not indicate the endosomal generation pathway, as small EVs (<150 nm) can also be generated by plasma membrane budding, and large EVs (>150 nm) can also be derived from the endosomal pathway. In addition, protein markers, such as CD9, CD63, flotillin and HSP90, are expressed on all EVs, and CD81 is expressed on sEVs, including both exosomes and microvesicles. Furthermore, TSG101 is mainly but not exclusively expressed on endosomal pathway-related sEVs (Thery et al., 2018). Regarding this, the current widely used isolation methods are not able to distinguish sEVs by their generation pathway; thus, we use the term sEVs to represent vesicles isolated from BMMSCs instead of “exosomes”. (Figure 1) We summarized the studies using BMMSC-derived sEVs as therapeutic agents in ischemic heart disease in recent years (Table 2) and found that sEVs have beneficial effects on IHD *via* their regenerative abilities and antiapoptotic and anti-inflammatory actions (Figure 1).

**TABLE 2:** Pre-clinical studies of using EVs derived from BMMSC in IHD.

Year	Animal model	Cell resource	EVs isolation separation	MSC-EVs size (nm)	EVs markers	Doses	Administration method	Effects	Ref
Promote angiogenesis									
2015	Myocardial infarction	Rat BMMSC	Exo-Quick-Tc Kit	50–100	CD63^+^	80 µg	Intramyocardial injection 60 min after ligation	Preserve cardiac function, reduced infarct size, enhancing the density of new capillary	Teng et al. (2015)
2018	Myocardial infarction	Mouse BMMSC	Exosome isolation Reagent	*NA*	CD63^+^, CD9^+^	600 µg	Intramyocardial injection after ligation	miR-132 overexpressed EVs promote angiogenesis and rescue cardiac function	Ma et al. (2018)
2019	STZ induced diabetic cardiomyopathy	Rat BMMSC	Exosome isolation kit	NA	CD63^+^	100 µg Once a week for 12 weeks	Intravenously injection	Downregulate TGF-β1 and Smad2, improve diabetes-induced cardiac fibrosis	Lin et al. (2019)
2020	Myocardial infarction	HIF-1α OE -Rat BMMSC	Ultracentrifugation 12 × 10^4^ g, 70 min	50–200	CD63^+^, TSG101^+^	2 × 10^10^ particles of EVs derived from MSC	Intramyocardial injection after ligation	Promoting neovessel formation, inhibiting fibrosis	Sun et al. (2020)
2020	Myocardial infarction	Mouse BMMSC	Exo-Quick-Tc Kit	Around 130	CD63^+^, CD9^+^,CD81^+^,TSG101^+^, Alix^+^, Hsp70^+^	.25 µmol of each	Intramyocardial injection after ligation	miR-19a/19b overexpressed EVs improve cardiac function and reduce cardiac fibrosis	Wang S et al. (2020)
2021	Ischemia 45 min-reperfusion 72 h	Hypoxia-Rat BMMSC	Ultracentrifugation 10 × 10^4^ g, 90 min	50–100	Alix^+^, TSG101^+^	3×10^11^ particles/100 µL	Caudal vein injection prior to reperfusion	Improved cardiac microvascular functions by reducing PDGFR-β levels at late stage of I/R	Wang et al. (2021)
Reduce apoptosis									
2017	Ischemia 30 min-reperfusion 2 h	Hypoxia-Rat BMMSC	Exosome isolation reagent	50–150	*NS*	5 µg	Intramyocardial injection 5 min prior to reperfusion	Improved cardiac function and reduced apoptosis	Liu et al. (2017)
2018	Myocardial infarction	Hypoxia-Mouse BMMSC	Ultracentrifugation 14 × 10^4^ g, 90 min	40–150	Alix^+^, TSG101^+^	10 μg/g, body weight	Intramyocardial injection after ligation	Inhibit cell apoptosis by delivering miR-125b-5p	Zhu et al. (2018)
2020	Ischemia 60 min-reperfusion 12 h	Mouse BMMSC	Exosome isolation reagent	Around 100	HSP70^+^, CD63^+^, CD9^+^	5 µg	Intramyocardial injection at ischemia 30 min s	Inhibit cell apoptosis and inflammation by delivering miR-25-3p and targeting pro-apoptotic proteins FASL/PTEN	Peng et al. (2020)
2020	Myocardial infarction	MIF overexpressed Human BMMSC	Exosome isolation Reagent	30–100	CD63^+^, CD81^+^	30 µg	Intramyocardial injection after ligation	Reduce infarct size, inhibit mitochondrial fragmentation and apoptosis	Liu et al. (2020)
2020	Myocardial infarction	Hypoxia-Rat BMMSC	Ultracentrifugation 11 × 10^4^ g, 75 min	30–150	CD63^+^, TSG101^+^	The EVs acquired from 1 × 10^6^ MSC	Intramyocardial injection after ligation	Protect cardiomyocyte apoptosis, miR-210/AIFM-3/AKT/P53	Cheng et al. (2020)
Reduce inflammation									
2015	Sepsis induced cardiac dysfunctin	Mouse- BMMSC	Ultracentrifuge 3.6 × 10^4^ g, 3 h	34–35	CD63^+^, CD81^+^	2 µg/per g, body weight	Caudal vein injection 1 h after CLP operation	Alleviated inflammation by delivery miR-223	Wang et al. (2015)
2018	Dox induced dilated cardiomyopathy	Mouse BMMSC	Ultracentrifugation 10 × 10^4^ g, 3 h	35	Alix^+^, TSG101^+^, CD9^+^, CD63^+^	300 µg	Caudal vein injection	Suppress cardiac inflammation	Sun et al. (2018)
2019	Ischemia 45min-reperfusion 3, 6, 12 days	Mouse BMMSC	Gradient centrifugation	50–150	CD9^+^, CD63^+^, TSG101^+^, Alix^+^	50 µg	Intramyocardial injection at reperfusion	Reduced infarct size, alleviated inflammation levels, containing miR-182/macrophage polarization	Zhao et al. (2019)
2021	Sepsis induced myocardial injury	Mouse BMMSC	Ultracentrifuge 10 × 10^4^ g, 4 h	Around 100	CD63^+^,CD9^+^	2 µg/per g, body weight	Caudal vein injection 1 h after CLP operation	Alleviated inflammation by miR-141/PTEN/β-catenin	Pei et al. (2021)
2022	Myocardial infarction	Mouse BMMSC	Ultracentrifugation 10 × 10^4^ g, 70 min	50–150	CD81^+^, TSG101^+^	15 µg once a week for 3 weeks	Caudal vein injection	Exosomal miR-129-5p protect hearts by targeting TRAT3/NF-κB	Yan et al. (2022)

AIFM, apoptosis inducing factor, mitochondria associated 3; CLP, cecalligation puncture; FASL, fas ligand; NF-κB, nuclear factor kappa-B; PTEN, phosphatase and tensin homolog deleted on chromosome ten; TRAT3, tumor necrosis factor receptor-associated factor 3.

## Regenerative effects of BMMSC-sEVs on IHD

A proteomic analysis study showed that sEVs derived from BMMSCs have a superior regenerative ability (Wang Z. G et al., 2020). The regenerative ability in hearts is reflected in promoting angiogenesis. Intramyocardial BMMSC-sEV injection enhanced the density of new functional capillaries and hence blood flow recovery in a rat myocardial infarction model (Teng et al., 2015). BMMSC-sEV treatment could increase the level of platelet-derived growth factor receptor-β (PDGFRβ), an angiogenetic factor, more than BMMSC treatment itself within 24 h after myocardial infarction in rats (Wang et al., 2021). PDGFRβ was enriched in fibrotic areas, although its expression was increased by BMMSC-sEV treatment at 24 h, but it was reduced after 4 weeks of myocardial infarction, which may be the reason for the antifibrotic effects of sEVs. Concurrently, modified cargoes of sEVs would enhance their effects. For example, overexpressed HIF-1α in sEVs results in better neovessel formation and fibrosis inhibitory functions, as well as higher expression levels of PDGF and VEGF compared to those of non-modified sEV treatment after myocardial infarction in rats (Sun et al., 2020). In addition, the beneficial functions of miRNAs in various diseases, including IHD have been investigated extensively (Zhang et al., 2018a; Zhang et al., 2018b; Liu et al., 2022), and miR-19a/19b-overexpressing sEVs combined with BMMSC therapy in the ischemic hearts of mice significantly enhanced the recovery of cardiac function and reduced cardiac fibrosis compared to non-transfected sEVs combined with BMMSCs (Wang S et al., 2020). miR-132 regulates endothelial cell behavior, and miR-132-overexpressing EVs in the ischemic hearts of mice markedly enhanced neovascularization in the peri-infarct zone and preserved heart functions (Ma et al., 2018). Moreover, the antifibrotic effects of EVs from BMMSCs were found in diabetic cardiomyopathy treatment (Lin et al., 2019), indicating that the regenerative ability of sEVs derived from BMMSCs is not specific to IHD.

## Anti-inflammatory actions of BMMSC-sEVs on IHD

The anti-inflammatory action of sEVs from BMMSCs is pivotal for their therapeutic effects in ischemic hearts. For example, in a mouse heart ischemia reperfusion injury model, sEVs derived from BMMSCs improved left ventricular ejection fraction (EF%) and fraction shortening (FS%), reduced infarct size, and alleviated the release of inflammatory factors (Zhao et al., 2019). In this study, miR-182 shuttling by sEVs targets Toll-like receptor 4 (TLR4), the inhibition of which leads to anti-inflammatory M2 macrophage conversion (Vergadi et al., 2017), thus promoting macrophage polarization and alleviating inflammation. Similarly, another study showed that in an ischemia-induced mouse heart failure model, sEVs derived from BMMSCs improved cardiac function by inhibiting NF-κB signaling, a transcription factor for cytokine release, and miR-129-5p carried by sEVs was proven to target tumor necrosis factor receptor-associated factor 3 (TRAF3), which subsequently regulates NF-kB (Yan et al., 2022). The anti-inflammatory effects of sEVs derived from BMMSCs were not only found in ischemic heart injury, doxorubicin-induced heart failure models and sepsis-induced heart failure models but also significantly reduced inflammatory factor release when BMMSC-derived sEVs are injected into hearts (Wang et al., 2015; Sun et al., 2018; Pei et al., 2021). In these studies, either the JAK pathway or the miR-141/miR-223 pathway was the major mediator of its anti-inflammatory effects (Table 2). In addition, sEVs have been shown to inhibit T-cell activation (Teng et al., 2015), which improves the microenvironment of the infarcted myocardium and contributes to angiogenesis and anti-inflammation.

## Antiapoptotic actions of BMMSC-sEVs on IHD

Apoptosis is a major pathological factor that causes heart failure after myocardial infarction. Apoptotic protein hyperactivation, insufficient autophagic activation and mitochondrial injury lead to cardiac cell apoptosis after myocardial infarction. sEVs derived from hypoxia-treated BMMSCs showed a decrease in the levels of several apoptosis-related genes, such as cleaved caspase-3, Bad and Bax, in the hearts of rats with myocardial infarction compared to controls. GW4869, which limits endosomal pathway EV formation, abolished the effects of EVs, showing that sEVs are responsible for their antiapoptotic effect (Cheng et al., 2020). Furthermore, in the study, the authors showed that miR-210 carried by sEVs could target the AIFM-3/AKT/p53 pathway (Cheng et al., 2020), which may be the core mechanism of protective effects, suggesting that the miRNAs carried by sEVs are the main reasons for its beneficial effects. Consistent with this finding, another study showed that sEVs derived from hypoxia-treated BMMSCs contained miR-125b-5p, which suppressed the expression of the proapoptotic genes p53 and BAK1 in cardiomyocytes, thus facilitating ischemic cardiac repair by ameliorating cardiomyocyte apoptosis (Zhu et al., 2018). In addition, in an ischemia reperfusion model, sEVs derived from BMMSCs decreased infarct size by delivering miR-25-3p, which directly targets and inhibits the proapoptotic proteins FASL/PTEN (Peng et al., 2020). Similarly, engineered sEVs with gene manipulation also showed antiapoptotic effects in IHD. For example, BMMSC EVs overexpressing macrophage migration inhibitory factor (MIF), a proinflammatory cytokine, enhanced heart function, reduced heart remodeling and reduced cardiomyocyte mitochondrial fragmentation, reactive oxygen species generation, and apoptosis compared to BMMSC EVs without MIF overexpression (Liu et al., 2020). Other conditions, such as sEVs derived from hypoxic BMMSCs, can reduce the myocardial infarction area and improve cardiac function by increasing autophagy levels (Liu et al., 2017), suggesting that appropriate modification of sEVs can enhance their antiapoptotic effects.

## Mitochondria containing in BMMSC-EVs

Whether EVs derived from BMMSC contain fully functional mitochondria is remain elusive. Because in clinical, subjects received allogenic bone marrow transplants detected almost no transfer of the donor mitochondria DNA (mtDNA) to the host mtDNA fraction in epithelial, connective, or skeletal muscle tissues, even exposure to the donor mtDNA in EV fractions for years (Tarnopolsky et al., 2020). In addition, studies showed that mitochondria mainly contain in larger EV (250 nm) rather than sEVs (100 nm), although EV (250 nm) contain all parts of mitochondria, their independent functionality inside EV cannot be confirmed due to methodological deficiencies (Wagner et al., 2022; Zorova et al., 2022).

Even then, mitochondrial transfer was shown to be important for the therapeutic effects of MSCs and MSC-EVs, in lung injury models, EVs derived from BMMSCs MSCs promote an anti-inflammatory and highly phagocytic macrophage phenotype through EV-mediated mitochondrial transfer (Morrison et al., 2017), as well as restore barrier integrity and normal levels of oxidative phosphorylation (Dutra Silva et al., 2021). These effects were also observed in renal ischemia reperfusion disease (Cao et al., 2020) and oculopathy (Jiang et al., 2020) with BMMSC-EVs treatment. Whether sEVs derived from BMMSC could transfer mitochondria to heart still need to be further investigated, but obesity induced adipocytes release sEVs (45–200 nm) contain oxidatively-damaged mitochondrial particles, which can be taken up by cardiomyocytes and they trigger a preconditioning environment, result in protects cardiomyocytes from acute oxidative stress (Kim et al., 2021). And EVs (>200 nm) derived from patient-specific induced pluripotent stem cell-derived cardiomyocytes (iCMs) mediate mitochondrial transfer mitigates DOX injury (O'Brien et al., 2021). Therefore, function of sEV-mitochondria or its components in cardiac diseases is a very interesting direction to explore.

## Current progress in improving BMMSC-sEV efficiency

Despite the benefits of EV-based therapy, low efficiency is the main obstacle preventing it from being used clinically. However, researchers are making progress in solving these problems. First, modified sEVs to overcome the poor homing efficiency have been investigated in several studies. One method is the modification of BMMSC-derived EVs with monocyte mimics through membrane fusion, which can be recruited by myocardial cells after MI (Zhang et al., 2020). The other way is to optimize the sEV delivery method; for example, a cardiac patch can dramatically enhance the retention of delivered substances, and an engineered EV spray, which later forms a stable gel patch on the heart, makes the therapy less invasive for cardiac patches (Yao et al., 2021). Second, genetically modified sEVs have shown good potential for IHD treatment. For example, using miR-455-3p, miR-30e, or miR-29c-transfected sEVs could protect myocardial infarction in rodent model (Li et al., 2020; Pu et al., 2021; Botello-Flores et al., 2022; Wang and Shen, 2022), as well as cardioprotective gene-transfected EVs, such as overexpress MIF (Liu et al., 2020), or FNDC5 (Deng et al., 2020), showed better therapeutic efficiency than unmodified EVs. Finally, EVs derived from some drug-pretreated MSCs can promote therapeutic effects; for example, atorvastatin-pretreated BMMSC-derived EVs enhanced therapeutic efficacy for the treatment of acute myocardial infarction by elevating lncRNA-H19 (Huang et al., 2020).

## Limitations and future directions

Although EVs derived from BMMSCs have shown promising therapeutic effects in heart injury, there are still some drawbacks that need to be solved. One issue is the route of injected EVs to the heart; most studies performed intramyocardial injection, while few studies performed caudal vein injection. The timing of injection was different according to the ischemia models (Table 2). For myocardial infarction treatment, EVs were mostly injected after ligation (Table 2), while for ischemia reperfusion injury treatment, EVs were administered at the middle time of ischemia or just prior to reperfusion. Another difference is the dose of EV administration in the preclinical experiments. The doses used in the IHD were diverse in each experiment, and they were independent of the species used in the experiments. For example, doses of 5 µg were used in ischemia reperfusion models of both mice and rats by intramyocardial injection; however, in another study, a dose of 50 µg was used in the same mouse model, and whether the dose difference is associated with the therapeutic outcome is unknown. Similarly, for myocardial infarction treatment, except for a few studies with multiple injections (once a week for several weeks), the total doses ranged from 30 μg to 600 µg in one injection, and a major difference in therapeutic effects was not observed. Of note, some studies use dose units other than micrograms, such as particles from BMMSCs or cell numbers of BMMSCs for their derived EVs, which makes it more difficult to identify the specific amount of EVs. In addition, different sEV populations can be obtained by different isolation methods. As shown in Table 2, sEV isolation from BMMSCs was conducted by either ultracentrifugation (>10 × 10^4^ g, >60 min) or a commercial exosome isolation kit (gradient centrifuge and filter). Six of eight studies that used the ultracentrifuge method to isolate sEVs verified the positive expression of TSG101 on the sEVs, but only 1 of the 10 studies that used a commercial kit for sEV isolation verified TSG101. Additionally, ultracentrifugation led to a sEV size of approximately 50–150 nm, and commercial kit-isolated sEVs were mostly approximately 50–100 nm. Whether these differences result in inconsistent outcomes of sEV therapy is unclear, but establishing a unified standard from extraction to quantity of sEVs is necessary to better evaluate their medicinal value. Lastly, although BMMSC have shown good therapeutic effects in IHD, accumulating evidence demonstrated that MSCs derived embryonic stem cell (ESC) or induced pluripotent stem cell (iPSC) exhibits superior therapeutic efficacy than BMMSCs in DOX induced cardiomyopathy (Zhang et al., 2015; Zhang et al., 2016), and in mouse limb ischemia disease (Lian et al., 2010), so ESC or iPSC can be served as an alternative source for BMMSC-sEV for IHD treatment.

Thus, BMMSC-derived sEVs showed potential therapeutic effects in IHD in preclinical studies, but the effectiveness of clinical application needs further research. In addition, further exploration is needed to optimize the quality control, dosage and method of administration of sEVs.
